# Urinary Tract Infections in Men in Primary Care in Catalonia, Spain

**DOI:** 10.3390/antibiotics12111611

**Published:** 2023-11-10

**Authors:** Silvia Fernández-García, Ana Moragas Moreno, Maria Giner-Soriano, Rosa Morros, Dan Ouchi, Ana García-Sangenís, Mònica Monteagudo, Ramon Monfà, Carl Llor

**Affiliations:** 1 Fundació Institut Universitari per a la Recerca a l’Atenció Primària de Salut Jordi Gol i Gurina, 08007 Barcelona, Spain; mginer@idiapjgol.info (M.G.-S.);; 2 Department of Medical Sciences, Universitat de Girona, 17004 Girona, Spain; 3 Universitat Autònoma de Barcelona, 08193 Bellaterra (Cerdanyola del Vallès), Spain; 4 Institut Català de la Salut, Center d’Atenció Primària Jaume I, 43005 Tarragona, Spain; 5 CIBER en Enfermedades Infecciosas Instituto Carlos III, 28029 Madrid, Spain; 6 Department of Medicine and Surgery, Universitat Rovira i Virgili, 43123 Reus, Spain; 7 Spanish Clinical Research Network, UIC IDIAPJGol, 08007 Barcelona, Spain; 8 Research Unit for General Practice, Department of Public Health, University of Southern Denmark, 5230 Odense, Denmark

**Keywords:** urinary tract infection, primary health care, male, anti-bacterial agents, diagnosis, electronic health records

## Abstract

Antimicrobial resistance is a major global problem that is primarily driven by the excessive and inappropriate utilization of antibiotics. Urinary tract infections (UTIs) are frequent in primary health care (PHC) and are typically treated with antibiotics. There is ample evidence on the management of this condition in women but not in men. The aim of this study was to describe the epidemiology of UTIs in men in Catalonia, Spain. We conducted a population-based observational cohort study that included male patients diagnosed with UTI within our SIDIAP and CMBD database during the period from 2012 to 2021. UTI diagnoses were grouped into five main groups (cystitis, prostatitis, orchitis and epididymitis, urethritis, and pyelonephritis). Of the 316,762 men with at least one recorded UTI episode, the majority were registered with a diagnosis of cystitis in PHC (212,958 patients). Quinolones were the most commonly recorded treatment for UTIs (between 18.3% and 38.6%, depending on the group), except for urethritis in which a combination of antibiotics (36.7%) was most frequently used. The treatment duration period was between 9 days and 18 days, except for the prostatitis group, in which treatment was extended to 21 days. Urine cultures were documented in up to 30% in the cystitis group. Pyelonephritis was the category linked to most septicemia cases (3.0%). Conclusions: This is the first study to assess UTIs in men using a large PHC database in Spain. The sociodemographic characteristics of our sample are similar to other studies in the literature. In our setting, the use of quinolones for the treatment of UTIs is the most registered, and its duration was between 9 days and 18 days, despite the fact that resistance to quinolones exceeds 20% of the strains in our area.

## 1. Introduction

It is estimated that by 2050, 10 million people worldwide may die from infections caused by multidrug-resistant pathogens if urgent actions are not taken to fight the problem of antimicrobial resistance, mainly among gram-negative bacteria, and particularly enterobacteria [1]. Antimicrobial resistance poses a challenge to both individual and public health. Escalating resistance can increase the duration of infections, heighten the risk of infecting others, and can result in elevated long-term expenses. Moreover, inappropriate use of antibiotics increases the frequency of associated, occasionally severe side effects and causes increased mortality [2,3]. Unnecessary use of antimicrobial agents is the main cause of resistance generation in the community, and it is estimated that 80% of antimicrobials are prescribed in primary health care (PHC) [4].

Urinary tract infections (UTIs) are a common infection in PHC, and most cases are treated with antibiotics [5,6,7]. UTI represents the second most frequent cause of antibiotic prescriptions in Spain [8]. UTIs are more common in women than in men, with almost 60% of women experiencing at least one episode during their lifetime [9,10]. In contrast, there are few studies on UTIs in the male population in PHC. A study from Sweden showed that lower UTIs are uncommon in men younger than 50 years, and pyelonephritis was more frequent in men older than 71 years [11]. In another study, the mean age of consultation of men with suspected UTI was 61 years, and the incidence increased with age [12].

In most cases, antibiotic treatment of uncomplicated UTIs is empirical, and the choice of antimicrobial is determined based on the most commonly implicated pathogen implicated and local resistance patterns. Several studies have suggested that up to 50% of antibiotic prescriptions are inappropriate [13,14]. Approximately 80% of uncomplicated UTI episodes are attributed to *Escherichia coli*, thereby requiring an antibacterial agent that effectively targets this pathogen [15,16,17]. Resistance of uropathogens to common antibiotics has increased significantly in recent years in Spain [18].

The diagnosis and treatment of UTIs in men in PHC vary compared to those in women. A recent systematic review highlighted the absence of a consensus regarding UTIs classification and treatment, which can differ from country to country [19]. The present guidelines in PHC in Spain recommend antibiotic treatment for 7 to 14 days, with the exception of acute prostatitis, which may require an extended treatment duration of up to 28 days [20,21].

The aim of our study was to describe the epidemiology of UTIs in men in Catalonia, Spain, between 2012 and 2021, using different local databases that provide us with information on clinical practice in PHC. This study is part of the project called “Urinary Tract Infections in Catalonia” (Infeccions del tracte urinari a Catalunya -ITUCAT-), which includes different work packages [22]. The main project is the European Union Electronic Register of Post-Authorization Studies EUPAS49724 [23].

## 2. Results

### 2.1. Population

Information was obtained from 316,762 episodes of UTI involving 2,305,703 men included in the databases between 2012 and 2021. The episodes were classified into five main groups: cystitis with 212,958 patients, prostatitis with 39,008 patients, orchitis and epididymitis with 31,180 patients, urethritis with 26,067 patients, and pyelonephritis with 7549 patients.

### 2.2. Sociodemographic Characteristics

The average age of men with UTIs ranged from 35 and 61 years. Men diagnosed with cystitis were older, while those with urethritis were younger (Table 1).

As shown in Table 1, more than half of the UTIs were diagnosed in primary care centers in urban settings. The cystitis group had the highest proportion of patients registered as residing in nursing homes, accounting for 7.1% of cases. Additionally, the MEDEA index, a measure of socio-economic deprivation by quintiles [24], was available, and no clear variations were observed among the various levels of socio-economic deprivation in this context.

### 2.3. Baseline Clinical Characteristics

The baseline clinical characteristics are described in Table 2.

Most of the population were smokers or ex-smokers, and this characteristic was observed in all UTI groups. The frequencies of alcohol consumption were similar in the groups evaluated. Likewise, the body mass index (BMI) values recorded in the different groups ranges from 26.1 to 28.4 kg/m^2^. The pyelonephritis group had more patients with obesity (BMI over 30%). The most frequent comorbidity not related to the urinary tract was respiratory system diseases in all evaluated groups, followed by dyslipidemia and diabetes mellitus. This trend was observed in all the UTI groups analyzed.

Regarding comorbidities related to the urinary tract, benign prostatic hyperplasia was the most frequently registered diagnosis in the cystitis group, and urinary lithiasis was the most frequently reported in the remaining groups.

### 2.4. Characteristics of UTI Episode

The frequency of presenting a subsequent episode of the same infection was low in all the groups analyzed (Table 3).

Among the evaluated infections, approximately 60% had documented antibiotic treatments. The cystitis group presented the highest rate of antibiotic registrations, accounting for 76.7% of cases in the database. The average duration of registered treatment was between 9 and 18 days, except in the prostatitis group, where the duration was 21 days.

Regarding the management of infections, urine cultures were most frequently requested in the cystitis and pyelonephritis groups, accounting for 35.1% and 29.5%, respectively. The record of urinary catheter use during the episode was low. In addition, the rate of referrals to specialized care was low, with the exception of the orchitis and epididymitis group, which had a referral rate of 22.9%. The pyelonephritis group had the highest incidence of septicemia (3.0%).

Table 4 provides a detailed overview of the antibiotic treatments recorded during UTI episodes, categorized by treatment group and specifying the most common antibiotic administered in each group. Quinolones were the predominant therapeutic category in all groups, except for the urethritis group. The most frequently prescribed antibiotic was ciprofloxacin. In contrast, antibiotic combinations were the most reported group in urethritis, with cefixime + azithromycin being the most common combination, followed by azithromycin + ciprofloxacin.

## 3. Discussion

This study describes the characteristics of UTIs in male patients, providing a significant contribution given the scarcity of information on this condition in comparison to women [11,19]. There is a dearth of clinical trials specifically conducted in men, posing challenges in terms of their management [25,26,27,28]. In addition, there is no consensus in the existing literature regarding the diagnosis of UTIs, with most articles categorizing it as a complicated UTI in men [19, 29, 30, 31, 32].

We found that, in general, UTIs in male patients in Catalonia tend to occur in middle-aged patients with a history of smoking, alcohol consumption, and overweight. In these patients, pathologies of the respiratory system were most frequently recorded, followed by dyslipidemia and diabetes mellitus in all groups. The most frequent urinary tract disorders in the UTI group were renal lithiasis, except for benign prostatic hypertrophy, which was more frequent in the cystitis group. The usual treatment in PHC for UTIs was a quinolone, especially ciprofloxacin, for periods ranging from 9 days to 18 days. Regarding the management of UTI, requests for urine cultures, the use of urinary catheters, and referral to specialized care during the infectious episode were infrequent. Few diagnoses of septicemia or renal failure were recorded in the UTIs.

The group with the highest number of diagnosed infections was cystitis. These patients were older, presenting more comorbidities related to the urinary system. It was also the group with the most records of antibiotic treatment during the episode of infection and with more requests for urine cultures. In the prostatitis group, longer treatment durations of up to 21 days were recorded, and in the orchitis and epididymitis groups, there were fewer requests for urine cultures and more referrals to hospitals. The urethritis group consisted of younger men, and the most commonly prescribed treatment was a combination of antibiotics, unlike the other UTIs. Finally, in the pyelonephritis group, there were fewer records of antibiotic treatment in patients with a higher percentage of renal lithiasis and urinary catheterization. In addition, it was the group in which more episodes of septicemia were recorded.

UTIs are typically diagnosed at an average age of 57 years, with the exception of cystitis, which tends to be diagnosed in an older population. This pattern mirrors observations in European countries, such as Norway or France [33,34], as well as in South African regions, such as Cape Town [29]. It should be noted that patients categorized in the orchitis and epididymitis, and the urethritis groups had lower average ages, as these conditions are commonly associated with sexually transmitted diseases that are more prevalent among younger age groups [20].

In our research, we found that men diagnosed with UTI were more likely to be smokers or former smokers. Additionally, they exhibited a moderate risk of alcohol consumption and overweight. Regarding tobacco use, a 2019 study revealed a positive correlation between UTI patients who were elderly, male, smokers (among other factors), and any cause of death within a 60 day period [14]. Nonetheless, further studies are necessary to assess this association.

Respiratory system diseases were the most commonly identified comorbidities, followed by dyslipidemia and diabetes mellitus. These same comorbidities were previously documented in a study conducted within our country in 2021 [35]. Diabetes mellitus is recognized as a risk factor for complicated UTI [20], although there is a lack of information regarding dyslipidemia in the literature. In terms of comorbidities associated with the urinary tract, benign prostatic hyperplasia was the most prevalent in the cystitis group. This comorbidity was also recorded in a French population sample, where 17.8% of patients with UTI had benign prostatic hyperplasia [34].

Approximately 60% of the UTIs sample had documented antibiotic treatment, although the literature indicates a varying range in both higher and lower percentages [11,13,30,33,34,36]. The pyelonephritis group showed the highest proportion of cases without recorded antibiotic treatment, a pattern consistent with findings in studies from Sweden and Norway [11,33]. This might be due to the nature of pyelonephritis, often managed as a high-risk condition with potential septicemia in hospital settings, where antibiotic treatment may not be registered in primary care records.

UTIs are usually treated with quinolones, with an average duration of 14 days. This trend in the use of fluoroquinolones has also been observed in PHC in France and Hungary [34,37]. However, in other countries, such as the UK and Sweden, the use of fluoroquinolones is lower [11,38], or UTIs are treated with other types of antibiotics, as in Norway [33]. Resistance rates of *Escherichia coli* remain below 5% against fosfomycin and nitrofurantoin in Catalonia, according to current resistance data. However, resistance against amoxicillin and clavulanic acid, as well as quinolones, currently exceeds 20% of strains [39]. Therefore, according to the records, antibiotic treatment is not in line with the resistance observed in our region.

Although the guidelines recommend requesting a urine culture in UTIs in men, our sample recorded a low request for urine culture, with the cystitis group having the highest record (30%). In contrast, in other countries, more urine cultures are performed during an episode of UTI [34].

In summary, according to the data analyzed, the sociodemographic characteristics of our sample are similar to the rest of the population observed in the literature. Likewise, we observed antibiotic use in UTI in men in PHC that would not be in line with the observed resistance rates.

One of the strengths of our study is the large sample size, which allowed us to obtain information from a large part of the male population of Catalonia. In addition, the information obtained corresponds to clinical practice in our primary care setting over a period of 10 years. On the other hand, the limitations of our study are those inherent to observational studies using data from electronic health records. Mainly, causality cannot be determined, and there may be biases due to confounding variables and problems in the records in the health registers. Another limitation of the study is that our data focused on primary settings. Our study provides information on the treatment and management of UTI in the male population in our setting, and this information is both important and useful for future research.

## 4. Materials and Methods

### 4.1. Study Design and Population

This was a population-based observational cohort study. The inclusion period was from 1 January 2012, to 31 December 2021. The study population consisted of male patients aged ≥18 years with a diagnosis of UTI registered in SIDIAP during the study period. UTI diagnoses were made according to the International Statistical Classification of Diseases and Related Health Problems, 10th Revision (ICD-10) codes and were grouped into 5 major groups, as shown in Table 5, to facilitate the assessment.

### 4.2. Data Collection and Data Sources

The data needed to carry out the project were obtained from the SIDIAP database, the Minimum Basic Data Sets (Conjunt minim bàsic de dades (CMBD)) of Hospital Discharges and Emergency Departments (Conjunt mínim bàsic de dades a d’hospitalizació d’aguts (CMBD-HA) and Conjunt mínim bàsic de dades d’atenció urgent (CMBD-UR), respectively) registries.

The SIDIAP contains pseudonymized clinical information from the Electronic Health Records in Primary Care (Estació clínica d’atenció primària) program [40], which is the electronic health record program for PHC of the Catalan Health Institute (Institut Català de la Salut (ICS)) in Catalonia. The ICS manages 328 PHC centers, covering a population of 5.8 million people (approximately 80% of the Catalan population) [41]. Information is available for more than 3384 health professionals who care for the adult population. The data recorded in SIDIAP contain sociodemographic data; health conditions, coded by ICD-10 [42]; clinical parameters; tobacco and alcohol use; diagnostic procedures; PHC laboratory test results; specialist referrals; and prescriptions of PHC medical staff, with the corresponding pharmacy invoice data, registered as anatomical, therapeutic, chemical (ATC) classification system codes [43]. Several reports have shown that SIDIAP data are useful for epidemiological research [41,44]. SIDIAP is listed in the European Network of Centers for Pharmacoepidemiology and Pharmacovigilance resources database [23].

The CMBD is a population-based registry that collects information on conditions treated in the health centers of Catalonia [45] and includes ICD-10 codes [43]. This registry contains information provided by all Catalan healthcare centers on healthcare activity and morbidity. The CMBD-HA contains information on acute hospitalizations, with reasons and dates for hospital admission, while the CMBD-UR reports activity in emergency departments.

### 4.3. Study Population

An initial population of 341,409 men with a record of at least one episode of UTI between 2012 and 2021 was obtained. Of this population, 5872 were children, and 335,537 were adults ≥ 18 years. Individuals were selected if they had been followed for at least one year after diagnosis, and thus the final sample was 316,762 men.

### 4.4. Variables and Outcomes

The study variables included sociodemographic information; clinical variables and health conditions, with ICD-10 codes; tobacco and alcohol consumption; PHC laboratory test requests; prescriptions, with their corresponding pharmacy invoice data registered as ATC codes [42]; CMBD-HA hospital information and CMBD-UR emergency department referral information.

For this study, an episode of UTI was defined as the date of the first diagnosis of UTI within a 14 day interval, regardless of the number of diagnoses recorded during this interval. Outside this interval, a diagnosis of UTI was considered a new episode of UTI. Furthermore, in order to define the parameters for antibiotic treatment, the request for PHC laboratory tests, referrals to specialized care, or the presence of a urinary catheter were deemed part of the same period of the UTI if they had been registered in the 15 days preceding the index date of the UTI and up to one month thereafter.

To describe the antibiotic therapies, categorization was conducted based on ATC groups, except for the beta-lactam antibacterial agents, for which only cephalosporins were included. Likewise, within the ATC category encompassing macrolides, lincosamides, and streptogramins, only macrolides were considered. Antibiotics discarded from the latter two categories were regrouped under ‘other antibacterials’. ATC groups not shown in Table 4 were included under the ‘other antibiotics’ group.

### 4.5. Statistical Analysis

The demographic and baseline characteristics of the participants were reported as frequencies and percentages for categorical variables and means and standard deviation or median and interquartile range for continuous variables, as appropriate. Patient characteristics were presented, stratified by groups based on UTI diagnoses. The distribution of antibiotic treatments among patient groups was ordered by frequency and by single or combination treatments.

### 4.6. Ethical Aspects and Data Confidentiality

The study was conducted in accordance with the Declaration of Helsinki, Good Research Practice principles and guidelines, and the Real Decreto 957/2020, of November 3, which regulates observational studies of medicines for human use. The study protocol was approved by the Ethics Committee of IDIAP Jordi Gol with ethical approval code 22/089-P (protocol code IJG-ITUCAT-2022, and date of approval: 27 July 2022).

## 5. Conclusions

We performed a large-scale study on UTI in men within the primary care setting. Almost the entire population of Catalonia was available, allowing the description of male patients with UTI.

The sociodemographic characteristics of our study are similar to other studies in the literature. In our setting, the use of quinolones for the treatment of UTI is the most commonly registered, and its duration was between 9 days and 18 days, despite the fact that the resistance against quinolones exceeds 20% of the strains in our area.

Nevertheless, our study has identified possible inadequate treatment patterns that need further research to improve treatment protocols and outcomes for UTIs in men.

## Figures and Tables

**Table 1:** Sociodemographic characteristics of patients diagnosed with UTI.

	Cystitis	Prostatitis	Orchitis and Epididymitis	Urethritis	Pyelonephritis
Number of patients	212,958	39,008	31,180	26,067	7549
Age ^1^ (mean (SD))	61.2 (18.9)	57.57 (15.3)	45.0 (17.0)	35.1 (12.8)	55.2 (17.1)
>80 years (%)	36,731 (17.2)	2474 (6.3)	947 (3.0)	134 (0.5)	579 (7.7)
Nursing home (%)	15,212 (7.1)	764 (2.0)	368 (1.2)	69 (0.3)	231 (3.1)
Area (%)					
Rural	37,020 (17.4)	6421 (16.5)	4824 (15.5)	2644 (10.1)	1171 (15.5)
Urban	138,985 (65.3)	26,983 (69.2)	21595 (69.3)	19,645 (75.4)	5264 (69.7)
MEDEA index ^2^ (%)					
U1	25,418 (11.9)	5534 (14.2)	3567 (11.4)	3500 (13.4)	943 (12.5)
U2	27,363 (12.8)	5629 (14.4)	4216 (13.5)	3692 (14.2)	1038 (13.8)
U3	29,323 (13.8)	5643 (14.5)	4481 (14.4)	3573 (13.7)	1106 (14.7)
U4	29,042 (13.6)	5370 (13.8)	4637 (14.9)	4065 (15.6)	1098 (14.5)
U5	27,839 (13.1)	4807 (12.3)	4694 (15.1)	4815 (18.5)	1079 (14.3)

^1^ Age in years. ^2^ MEDEA index was categorized in five quintiles: U1 least deprived until U5 most deprived.

**Table 2:** Baseline clinical characteristics of patients diagnosed with UTI.

	Cystitis	Prostatitis	Orchitis and Epididymitis	Urethritis	Pyelonephritis
Number of patients	212,958	39,008	31,180	26,067	7549
Smoking status ^1^ (%)					
Non-smoker	5791 (18.1)	1257 (21.0)	1214 (26.6)	1374 (35.2)	253 (22.3)
Ex-smoker	11,747 (36.8)	1994 (33.4)	1843 (40.4)	1691 (43.3)	391 (34.4)
Smoker	14,425 (45.1)	2728 (45.6)	1501 (32.9)	843 (21.6)	493 (43.4)
Alcohol ^1^ (%)					
No risk	64,832 (49.3)	9601 (42.4)	6137 (43.1)	3693 (40.3)	2021 (48.0)
Moderate risk	63,225 (48.0)	12,379 (54.7)	7578 (53.2)	5074 (55.4)	2055 (48.8)
High risk	3530 (2.7)	656 (2.9)	521 (3.7)	388 (4.2)	138 (3.3)
Body Mass Index ^1,2^ (mean (SD))	28.31 (4.7)	28.45 (4.5)	27.88 (4.9)	26.10 (4.6)	28.43 (4.9)
Body Mass Index > 30 (%) ^3^	38,212 (31.4)	6628 (31.7)	3499 (28.2)	1236 (17.0)	1268 (33.0)
Comorbidities no related to urinary tract					
Diabetes mellitus (%)	19,552 (9.2)	2091 (5.4)	884 (2.8)	251 (1.0)	560 (7.4)
Dyslipidemia (%)	28,565 (13.4)	3464 (8.9)	1571 (5.0)	376 (1.4)	772 (10.2)
Diseases of the nervous system (%)	7487 (3.5)	487 (1.2)	229 (0.7)	52 (0.2)	126 (1.7)
Cerebrovascular diseases (%)	9752 (4.6)	952 (2.4)	360 (1.2)	79 (0.3)	218 (2.9)
Diseases of the respiratory system (%)	52,215 (24.5)	8278 (21.2)	5671 (18.2)	3920 (15.0)	1652 (21.9)
Musculoskeletal system, and connective tissue diseases (%)	2530 (1.2)	301 (0.8)	108 (0.3)	48 (0.2)	98 (1.3)
Diseases of the digestive system (%)	5212 (2.4)	610 (1.6)	357 (1.1)	124 (0.5)	186 (2.5)
Comorbidities related to urinary tract					
Benign prostatic hypertrophy (%)	18,185 (8.5)	2010 (5.2)	924 (3.0)	127 (0.5)	394 (5.2)
Urinary lithiasis (%)	14,668 (6.9)	2273 (5.8)	1375 (4.4)	775 (3.0)	843 (11.2)
Chronic renal insufficiency (%)	8103 (3.8)	633 (1.6)	245 (0.8)	44 (0.2)	256 (3.4)

^1^ Variables with missing data. ^2^ Values of body mass index (kg/m^2^). ^3^ Obesity by body mass index over 30.

**Table 3:** Characteristics of UTI episode.

	Cystitis	Prostatitis	Orchitis and Epididymitis	Urethritis	Pyelonephritis
Number of patients	212,958	39,008	31,180	26,067	7549
Episodes ^1^ (%)					
1 episode	144,909 (68.0)	33,455 (85.8)	28,506 (91.4)	22,947 (88.0)	6771 (89.7)
2 episodes	37,611 (17.7)	4408 (11.3)	2290 (7.3)	2437 (9.3)	639 (8.5)
3 or more episodes	30,438 (14.3)	1145 (2.9)	384 (1.2)	683 (2.6)	139 (1.8)
Follow-up—years ^2^ (mean (SD))	5.5 (2.9)	6.1 (2.9)	6.4 (2.9)	5.9 (2.8)	6.1 (2.9)
Median [IQR]	5.1 [3.0, 8.0]	6.0 [3.6, 8.5]	6.3 [3.9, 8.9]	5.5 [3.5, 8.2]	5.9 [3.6, 8.4]
Treatment episodes (%)					
Unknown	49,555 (23.3)	14,363 (36.8)	12,175 (39.0)	7075 (27.1)	3038 (40.2)
1 Antibiotic	122,922 (57.7)	18,156 (46.5)	14,429 (46.3)	9035 (34.7)	3212 (42.5)
2 Antibiotics	31,343 (14.7)	5045 (12.9)	3621 (11.6)	8217 (31.5)	963 (12.8)
>2 Antibiotics	9138 (4.3)	1444 (3.7)	955 (3.1)	1740 (6.7)	336 (4.5)
Time under treatment—days (mean (SD))	13.9 (41.0)	21.2 (38.1)	14.4 (29.7)	9.5 (26.2)	18.2 (44.8)
Median—days [IQR]	9.0 [7.0, 14.0]	20.0 [11.0, 27.0]	10.0 [7.0, 16.0]	7.0 [1.0, 10.0]	14.0 [10.0, 21.0]
Urine culture (%)	74,829 (35.1)	10,103 (25.9)	4124 (13.2)	3845 (14.8)	2225 (29.5)
Urinary catheter (%)	4323 (2.0)	774 (2.0)	83 (0.3)	40 (0.2)	288 (3.8)
Septicemia (%)	1349 (0.6)	272 (0.7)	31 (0.1)	197 (0.8)	230 (3.0)
Specialized care referrals ^3^ (%)	16,977 (8.0)	5757 (14.8)	7149 (22.9)	1722 (6.6)	1222 (16.2)

^1^ Number of recorded episodes of the group. More than two episodes represent the number of subsequent episodes. ^2^ Time between first diagnosis and death or end of follow-up. ^3^ Registration of specialized care referrals from primary care.

**Table 4:** Antibiotic treatment of UTI.

	Cystitis	Prostatitis	Orchitis and Epididymitis	Urethritis	Pyelonephritis
Treatment group (%)					
Quinolones	76,726 (36.0)	15,042 (38.6)	11,061 (35.5)	1524 (5.8)	1381 (18.3)
Ciprofloxacin	61,808 (80.6)	13,460 (89.5)	8434 (76.2)	1232 (80.8)	1213 (87.8)
Penicillin	23,088 (10.8)	2141 (5.5)	2953 (9.5)	593 (2.3)	1044 (13.8)
Amoxicillin	22,447 (97.2)	2079 (97.1)	2864 (97.0)	537 (90.6)	1023 (98.0)
Cephalosporins	12,605 (5.9)	2695 (6.9)	1157 (3.7)	430 (1.6)	1042 (13.8)
Cefuroxime	9164 (72.7)	1636 (60.7)	799 (69.1)	96 (22.3)	666 (63.9)
Cefixime	3018 (23.9)	789 (29.3)	305 (26.4)	167 (38.8)	299 (28.7)
Quinolones + Penicillin	7116 (3.3)	944 (2.4)	738 (2.4)	89 (0.3)	218 (2.9)
Amoxicillin + ciprofloxacin	5425 (76.2)	828 (87.7)	583 (79.0)	69 (77.5)	183 (83.9)
Fosfomycin	17,596 (8.3)	260 (0.7)	70 (0.2)	136 (0.5)	45 (0.6)
Macrolides	847 (0.4)	139 (0.4)	260 (0.8)	3857 (14.8)	17 (0.2)
Azithromycin	710 (83.8)	109 (78.4)	229 (88.1)	3795 (98.4)	<5%
Other antibacterials ^1,2^	3731 (1.8)	667 (1.7)	686 (2.2)	2784 (10.7)	77 (1.0)
Doxycycline	264 (7.1)	49 (7.35)	501 (73.0)	2679 (96.2)	<5%
Trimethoprim + sulfamethoxazole	1868 (50.1)	513 (76.9)	131 (19.1)	<5%	55 (71.4)
Other combinations ^2^	21,694 (10.2)	2757 (7.1)	2080 (6.7)	9579 (36.7)	687 (9.1)
Amoxicillin + cefuroxime	<5%	<5%	<5%	<5%	77 (11.2)
Cefuroxime + ciprofloxacin	2069 (9.5)	424 (15.4)	160 (7.7)	<5%	76 (11.1)
Cefixime + azithromycin	<5%	<5%	<5%	1627 (17.0)	<5%
Azithromycin + ciprofloxacin	<5%	<5%	203 (9.8)	1594 (16.6)	<5%
Ciprofloxacin + fosfomycin	3061 (14.1)	181 (6.6)	<5%	<5%	<5%

^1^ Included the ATC group of beta-lactam antibacterial agents (except cephalosporins), lincosamides, and streptogramins. ^2^ Only the most frequently registered combinations are listed.

**Table 5:** Health problems included in the study, coded by ICD-10.

Groups ^1^	ICD—10 ^2^	Health Problems
Pyelonephritis	N10	Acute tubulointerstitial nephritis
Cystitis	N30	Cystitis
	N30.0	Acute cystitis
	N30.1	Interstitial cystitis (chronic)
	N30.2	Other chronic cystitis
	N30.3	Trigonitis
	N30.8	Other cystitis
	N30.9	Cystitis, unspecified
	N39	Urinary tract infection, site not specified
Urethritis	N34.1	Nonspecific urethritis
	N34.3	Urethral syndrome, unspecified
Prostatitis	N41.0	Acute prostatitis
	N41.1	Chronic prostatitis
	N41.3	Prostatocystitis
Orchitis and epididymitis	N45.0	Orchitis, epididymitis, and epididymo-orchitis with abscess
	N45.9	Orchitis, epididymitis, and epididymo-orchitis without abscess

^1^ Groups created for the assessment of UTIs. ^2^ Codes from the International Statistical Classification of Diseases and Related Health Problems, 10th Revision.

## Data Availability

The data are not publicly available due to national legal regulations.

## References

[1] O’Neill J. (2014). Review on Antimicrobial Resistance. Antimicrobial Resistance: Tackling a Crisis for the Health and Wealth of Nations.

[2] Murray C.J., Ikuta K.S., Sharara F., Swetschinski L., Robles Aguilar G., Gray A., Han C., Bisignano C., Rao P., Wool E. (2022). Global Burden of Bacterial Antimicrobial Resistance in 2019: A Systematic Analysis. Lancet.

[3] World Health Organization (WHO) Antibiotic Resistance. https://www.who.int/news-room/fact-sheets/detail/antibiotic-resistance.

[4] Hay D. (2019). Antibiotic Prescribing in Primary Care. BMJ.

[5] Tandogdu Z., Wagenlehner F.M.E. (2016). Global Epidemiology of Urinary Tract Infections. Curr. Opin. Infect. Dis..

[6] Malmartel A., Ghasarossian C. (2016). Epidemiology of Urinary Tract Infections, Bacterial Species and Resistances in Primary Care in France. Eur. J. Clin. Microbiol. Infect. Dis..

[7] Kornfält Isberg H., Melander E., Hedin K., Mölstad S., Beckman A. (2019). Uncomplicated Urinary Tract Infections in Swedish Primary Care; Etiology, Resistance and Treatment. BMC Infect. Dis..

[8] Llor C., Hernández S. (2010). Enfermedad Infecciosa En Atención Primaria: Estudio Prospectivo Efectuado Durante Todo Un Año. Enfermedades Infecc. Microbiol. Clín..

[9] Foxman B. (2002). Epidemiology of Urinary Tract Infections: Incidence, Morbidity, and Economic Costs. Am. J. Med..

[10] Fihn S.D. (2003). Acute Uncomplicated Urinary Tract Infection in Women. N. Engl. J. Med..

[11] Kornfält Isberg H., Hedin K., Melander E., Mölstad S., Beckman A. (2019). Increased Adherence to Treatment Guidelines in Patients with Urinary Tract Infection in Primary Care: A Retrospective Study. PLoS ONE.

[12] Hummers-Pradier E., Ohse A.M., Koch M., Heizmann W.R., Kochen M.M. (2004). Urinary Tract Infection in Men. Int. J. Clin. Pharmacol. Ther..

[13] Gharbi M., Drysdale J.H., Lishman H., Goudie R., Molokhia M., Johnson A.P., Holmes A.H., Aylin P. (2019). Antibiotic Management of Urinary Tract Infection in Elderly Patients in Primary Care and Its Association with Bloodstream Infections and All Cause Mortality: Population Based Cohort Study. BMJ.

[14] Shallcross L., Rockenschaub P., Blackburn R., Nazareth I., Freemantle N., Hayward A. (2020). Antibiotic Prescribing for Lower UTI in Elderly Patients in Primary Care and Risk of Bloodstream Infection: A Cohort Study Using Electronic Health Records in England. PLoS Med..

[15] de Cueto M., Aliaga L., Alós J.I., Canut A., Los-Arcos I., Martínez J.A., Mensa J., Pintado V., Rodriguez-Pardo D., Yuste J.R. (2017). Resumen Ejecutivo Del Diagnóstico y Tratamiento de Las Infecciones Del Tracto Urinario. Guía de La Sociedad Española de Enfermedades Infecciosas y Microbiología Clínica (SEIMC). Enfermedades Infecc. Microbiol. Clín..

[16] Palou J., Pigrau C., Molina I., Ledesma J.M., Angulo J. (2011). Etiología y Sensibilidad de Los Uropatógenos Identificados En Infecciones Urinarias Bajas No Complicadas de La Mujer (Estudio ARESC): Implicaciones En La Terapia Empírica. Med. Clin..

[17] Rodriguez-Mañas L. (2020). Urinary Tract Infections in the Elderly: A Review of Disease Characteristics and Current Treatment Options. Drugs Context.

[18] European Centre for Disease Prevention and Control (2022). Antimicrobial Resistance in the Eu/EEA (EARS-Net)—Annual Epidemiological Report 2020.

[19] Soudais B., Ribeaucoup F., Schuers M. (2023). Guidelines for the Management of Male Urinary Tract Infections in Primary Care: A Lack of International Consensus-a Systematic Review of the Literature. Fam. Pract..

[20] Patología Infecciosa en: Grupo de Trabajo de Enfermedades Infecciosas de la semFYC (2017). Manual de Enfermedades Infecciosas En Atención Primaria.

[21] (2018). Guía de Terapéutica Antimicrobiana Del Área Aljarafe.

[22] Moreno A.M., Fernández-García S., Llor C., Ouchi D., García-Sangenís A., Monteagudo M., Monfà R., Giner-Soriano M. (2023). Diagnostic and Therapeutic Management of Urinary Tract Infections in Catalonia, Spain: Protocol for an Observational Cohort Study. JMIR Res. Protoc..

[23] European Network of Centres for Pharmacoepidemiology and Pharmacovigilance ENCePP Resources Database. https://www.encepp.eu/encepp/resourcesDatabase.jsp.

[24] Domínguez-Berjón M.F., Borrell C., Cano-Serral G., Esnaola S., Nolasco A., Pasarín M.I., Ramis R., Saurina C., Escolar-Pujolar A., Correspondencia M. (2008). Constructing a Deprivation Index Based on Census Data in Large Spanish Cities [the MEDEA Project]. Gac. Sanit..

[25] Mospan G.A., Wargo K.A. (2016). 5-Day versus 10-Day Course of Fluoroquinolones in Outpatient Males with a Urinary Tract Infection (UTI). J. Am. Board Fam. Med..

[26] Van Nieuwkoop C., van der Starre W.E., Stalenhoef J.E., van Aartrijk A.M., van der Reijden T.J.K., Vollaard A.M., Delfos N.M., van ’t Wout J.W., Blom J.W., Spelt I.C. (2017). Treatment Duration of Febrile Urinary Tract Infection: A Pragmatic Randomized, Double-Blind, Placebo-Controlled Non-Inferiority Trial in Men and Women. BMC Med..

[27] Drekonja D.M., Trautner B., Amundson C., Kuskowski M., Johnson J.R. (2021). Effect of 7 vs 14 Days of Antibiotic Therapy on Resolution of Symptoms among Afebrile Men with Urinary Tract Infection: A Randomized Clinical Trial. JAMA J. Am. Med. Assoc..

[28] Farrell K., Tandan M., Santiago V.H., Gagyor I., Braend A.M., Skow M., Vik I., Jansaaker F., Hayward G., Vellinga A. (2021). Treatment of Uncomplicated UTI in Males: A Systematic Review of the Literature. BJGP Open.

[29] Keuler N., Johnson Y., Coetzee R. (2022). Treating Urinary Tract Infections in Public Sector Primary Healthcare Facilities in Cape Town, South Africa: A Pharmaceutical Perspective. S. Afr. Med. J..

[30] Plate A., Kronenberg A., Risch M., Mueller Y., Di Gangi S., Rosemann T., Senn O. (2020). Treatment of Urinary Tract Infections in Swiss Primary Care: Quality and Determinants of Antibiotic Prescribing. BMC Fam. Pract..

[31] Plate A., Kronenberg A., Risch M., Mueller Y., Di Gangi S., Rosemann T., Senn O. (2019). Active Surveillance of Antibiotic Resistance Patterns in Urinary Tract Infections in Primary Care in Switzerland. Infection.

[32] Johansen T.E.B., Botto H., Cek M., Grabe M., Tenke P., Wagenlehner F.M.E., Naber K.G. (2011). Critical Review of Current Definitions of Urinary Tract Infections and Proposal of an EAU/ESIU Classification System. Int. J. Antimicrob. Agents.

[33] Haugom L.E.A., Ruths S., Emberland K.E., Eliassen K.E.R., Rortveit G., Wensaas K.A. (2021). Consultations and Antibiotic Treatment for Urinary Tract Infections in Norwegian Primary Care 2006–2015, a Registry-Based Study. BMC Fam. Pract..

[34] Soudais B., Lacroix-Hugues V., Meunier F., Gillibert A., Darmon D., Schuers M. (2021). Diagnosis and Management of Male Urinary Tract Infections: A Need for New Guidelines. Study from a French General Practice Electronic Database. Fam. Pract..

[35] Ramos Lázaro J., Chico C., Jove N., Blázquez Fernández A.B., del Fernández Monasterio M.M., Smithson A. (2021). Tratamiento Antimicrobiano Domiciliario Endovenoso En Hombres Con Infección Del Tracto Urinario Febril: Diferencias Entre El Modelo de Evitación de Ingreso y El de Alta Precoz Hospitalaria. Outpatient Intravenous Antimicrobial Therapy in Men with Febrile Urinary Tract Infecionts: Differences between the Hospital Admissión-Avoidance and Early-Discharge Models. Emergencias.

[36] Spek M., Cals J.W.L., Oudhuis G.J., Savelkoul P.H.M., de Bont E.G.P.M. (2020). Workload, Diagnostic Work-up and Treatment of Urinary Tract Infections in Adults during out-of-Hours Primary Care: A Retrospective Cohort Study. BMC Fam. Pract..

[37] Benko R., Matuz M., Juhasz Z., Bognar J., Bordas R., Soos G., Hajdu E., Peto Z. (2019). Treatment of Cystitis by Hungarian General Practitioners: A Prospective Observational Study. Front. Pharmacol..

[38] Ahmed H., Farewell D., Jones H.M., Francis N.A., Paranjothy S., Butler C.C. (2018). Incidence and Antibiotic Prescribing for Clinically Diagnosed Urinary Tract Infection in Older Adults in UK Primary Care, 2004–2014. PLoS ONE.

[39] Badia J.M., Barrufet P., Calbo E., Besoli A., Casas I., Diaz E., Domenech D., Duran J., Gasch O., Grau S. (2022). Vigilància de Les Infeccions Relacionades Amb l’Atenció Sanitària de Catalunya (VINCat): Informe Anual 2022.

[40] Information System for Research in Primary Care. https://www.sidiap.org/index.php/en/.

[41] Recalde M., Rodríguez C., Burn E., Far M., García D., Carrere-Molina J., Benítez M., Moleras A., Pistillo A., Bolíbar B. (2022). Data Resource Profile: The Information System for Research in Primary Care (SIDIAP). Int. J. Epidemiol..

[42] World Health Organization International Statistical Classification of Diseases and Related Health Problems 10th Revision. https://apps.who.int/iris/handle/10665/246208.

[43] World Health Organization ATC/DDD Index 2023. https://www.whocc.no/atc_ddd_index/.

[44] Bolíbar B., Fina Avilés F., Morros R., Del Mar Garcia-Gil M., Hermosilla E., Ramos R., Rosell M., Rodríguez J., Medina M., Calero S. (2012). Base de Datos SIDIAP: La Historia Clínica Informatizada de Atención Primaria Como Fuente de Información Para La Investigación Epidemiológica. Med. Clin..

[45] Servei Català de la Salut Conjunt Mínim Bàsic de Dades (CMBD). https://catsalut.gencat.cat/ca/proveidors-professionals/registres-catalegs/registres/cmbd/.

